# Epidemic outbreak of Chikungunya in two neighboring towns in the Colombian Caribbean: a survival analysis

**DOI:** 10.1186/s13690-016-0169-1

**Published:** 2017-01-06

**Authors:** Misael Oviedo-Pastrana, Nelson Méndez, Salim Mattar, Germán Arrieta, Luty Gomezcaceres

**Affiliations:** 1 Universidad de Córdoba, Instituto de Investigaciones Biológicas del Trópico, Carrera 6 # 76-103, Montería, Córdoba Colombia; 2 Corporación Universitaria del Caribe (CECAR), Grupo de Salud Pública, Km 1, vía Corozal, Sincelejo, Colombia; 3 Clínica Salud Social SAS, Carrera 16 # 27A-74, Sincelejo, Colombia

**Keywords:** Kaplan meier estimate, Environment and public health, Comparative study, Infectious disease vector, Health economics, Culicidae, Risk management, Demography

## Abstract

**Background:**

The first autochthonous Chikungunya virus transmission in Colombia was reported in September 2014. Three months later, every town in the Caribbean region was affected, including the bordering towns of Ovejas and Corozal, in the department of Sucre. The objective of the study was to analyze and compare the temporal dynamics of the outbreak of Chikungunya in two towns of the department of Sucre.

**Methods:**

Households with suspicious cases with clinical symptomatology for Chikungunya were enrolled. In each house an epidemiological questionnaire was applied to collect economic and social information and methods for vector control.

**Results:**

The study analyzed data collected between 09/01/2014 and 01/31/2015; 458 families in Corozal and 516 families in Ovejas were identified with Chikungunya cases. Estimated attack rates were 10,621 cases and 1640 cases per 100,000 inhabitants, in Ovejas and Corozal, respectively. The 75-day survival curve was 27.2% lower (0.632, CI = 0.614–0.651) in Ovejas than in Corozal (0.904, CI = 0.891–0.917). After 120 days, both curves showed a stable horizontal slope, close to a survival probability of 0.54, indicating the end of the epidemic period. The log-rank test (*X* ^2^ = 94.6, 1fd, *p*-value = 0.000) showed the improved survival of Chikungunya in the town of Corozal. The relative risk between the two towns was 0.863 (CI = 0.809–0.921; *p*-value < 0.001).

**Conclusions:**

The dynamics of the temporal distribution of CHIKV could be influenced by socioeconomic and preventable risk factors. Poor socioeconomic conditions such as the lack and poor efficiency of water supply and waste collection services could be determining factors in the proliferation of CHIKV. The survival analysis proved to be a suitable method for studying the presentation of CHIKV and can be applied to other prevalent vector-borne diseases such as the ZIKA and Dengue.

## Background

Chikungunya virus (CHIKV) is an alphavirus of the *Togaviridae* family, the etiologic agent of an acute febrile illness that presents with myalgia, rash and severe joint pain, which can be debilitating and persist for months or years [1, 2]. CHIKV generates an excess morbidity that overloads the public health systems [3, 4].

The arrival of CHIKV into the Americas is fairly recent, and the first autochthonous transmission was reported in December 2013 in the island of St. Martin [5, 6]. Currently in the Americas, transmission of the Asian genotype CHIKV [7–9] and East-Central-South African (ECSA) genotype [10] has been reported. According to reports of suspected and confirmed cases of CHIKV in America, the Pan American Health Organization [11] reported more than one million cases in the year 2014. In 2015, 666,311 cases were reported, 54% of which (359,728 cases) were reported in Colombia [12]. In 2016, until epidemiological week #28, 9% of 195,628 reported cases were from Colombia (17.898 cases) [13]. The real burden of disease is underestimated; and an important percentage of underreporting of cases is probably due to the nonattendance of sick people to health centers [14].

In Colombia, the first case of autochthonous transmission of CHIKV took place in September of 2014 in the town of Mahates, Bolivar [15]. Three months later, by epidemiological week #53, 96,687 cases had been reported, 66,118 (68.4%) of which belonged to the Caribbean region and 13,464 (13.9%) were from the department of Sucre. Of the eight probable deaths reports due to CHIKV, two were reported in the department of Sucre, one in Corozal and another in the town of Sincelejo [16]. The spread of the disease can be attributed to the mobilization of people (with viremia or in incubation period) into nearby geographical areas; the towns near main roads have been systematically affected.

The high susceptibility of the Colombian population to CHIKV and the high prevalence of *Aedes aegypti* have generated a complex epidemiological framework [17], a large number of cases are still being reported with the concomitant high economic and social impact, albeit a marked underreporting trend. The combination of Dengue, Chikungunya and Zika virus complicates the public health problem in the Colombian population where problems due to poverty, poor basic sanitation and poor vector control persist.

Knowledge and follow up regarding the evolution of outbreaks and the identification of causal factors can contribute to the implementation of more effective measures against the disease. Statistical methods such as the survival analysis can be used to understand the temporal variation of any event of interest in public health [18]; including, the temporal dynamics of infectious diseases transmitted by vectors.

The objective of the study was to analyze the temporal evolution between 2014 and 2015 of an epidemic outbreak of Chikungunya in two neighbouring towns in the Caribbean region of Colombia.

## Methods

### Type of study, area and population

**Fig. 1:**
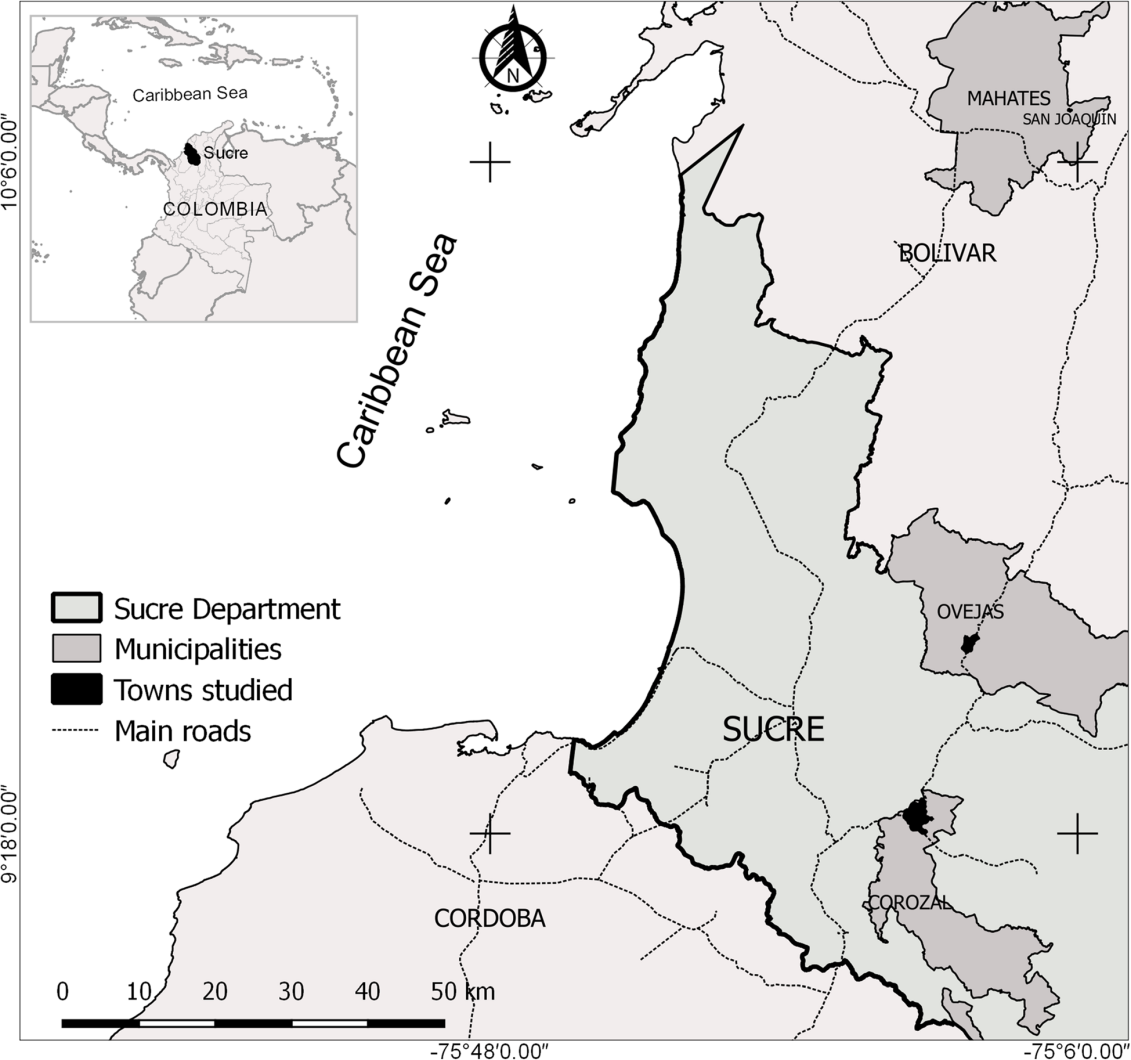
Study area in the towns of Corozal and Ovejas, in the department of Sucre, Colombia

A retrospective study was conducted to describe and analyze the demographic, socioeconomic and epidemiological characteristics of the population in two neighbouring towns affected by an outbreak of Chikungunya. The study area included the urban area of the town of Corozal and Ovejas, in the department of Sucre, Colombia (Fig. 1). The towns of Ovejas and Corozal are separated by 22 km; they have average temperatures of 28 °C and an altitude of 265 and 174 m above sea level, respectively. The areas were selected because of their proximity and because both showed a CHIKV outbreak emergent at the end of 2014. In addition, both are relatively near the town of Mahates, where it likely appeared to the first autochthonous case of this disease in the country [8, 15]. In 2015, the urban population in Ovejas and in Corozal was estimated at 11,947 and 51,157 inhabitants, respectively [19].

We performed convenience sampling in the two chosen populations, selecting the areas of low socioeconomic level and with environmental conditions favourable to mosquito proliferation, which include proximity to water reservoirs and the accumulation of wastes. In each selected site, households were selected if any of its members had Chikungunya in the previous months and their story matched with the definition for a clinical suspect case from the Colombian National Institute of Health [20].

An epidemiological questionnaire was applied on 01/31/2015 in each selected household. The relevant aspects in the questionnaire included: characteristics of the house, composition of the household, family income, medical care and health expenses due to Chikungunya; public utilities available and predisposing factors associated with the disease; preventive measures to reduce the proliferation and bites of mosquitoes. Potential cases were questioned regarding the beginning of the symptoms of the disease and other clinical aspects.

### Data analysis

Data was collected in a spreadsheet using Microsoft Excel®. Frequencies, summary statistics and other descriptive analyses were calculated for the variables under study using the statistical software EpiInfo™, version 7.2.0.1. A *p*-value < 0.05 was considered statistically significant. Rates of incidence, epidemic curves and survival curves were calculated to analyze the temporal evolution of the outbreak of Chikungunya in the two neighbouring towns affected. Survival analysis used the Kaplan-Meier model [21], which is a non-parametric, analytical and graphic technique that estimates the probability of survival by the maximum likelihood method; the response variable is the time of survival or time-to-event, corresponding to the time elapsed since the initial moment until the occurrence of a certain event; when the event of interest is not observed it is defined as censoring.

In this study the epidemiological event was the presence of clinically suspected cases of Chikungunya and the survival (time-to-event) time was the time it took people to get sick from the appearance of the first case of Chikungunya in the two towns. The Kaplan-Meier estimator was applied using the Survival Analysis package in the statistical software R.

For the survival analysis an additional database was built in which each affected individual living in the houses was characterized according to his/her status for censoring, time of disease and town to which he/she belongs. Individuals who were not ill were considered to be censored data. The log-rank test was used to compare the curves of Chikungunya survival in the towns of Ovejas and Corozal; this test calculate the Chi-square for each time of each event in each group, adds the results and compare between them [18]. In addition, and through contingency tables assessed the possible association between socioeconomic factors and preventive measures that could explain the differences between the survival curves of the two towns.

## Results

### Affected population and incidence of Chikungunya

The study analyzed data obtained between 09/01/2014 and 01/31/2015. The date of the beginning of the outbreak in the town of Corozal was on 09/05/2014 and in the town of Ovejas on 09/15/2014.

We reported 458 families affected by Chikungunya in Corozal including 1995 residents, of which 839 (42%) became ill. Similarly, we reported 516 families affected in Ovejas, identifying 2605 residents with high risk of exposure, of which 1269 (49%) became ill. An estimated relative risk of 0.863 (CI = 0.809–0.921; *p*-value <0.001) suggested a protective factor for Chikungunya for the inhabitants of Corozal in relation to the inhabitants of Ovejas.

**Fig. 2:**
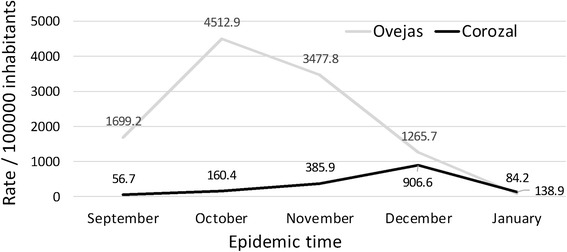
Monthly incidences rates of chikungunya in the towns of Corozal and Ovejas

The attack rate in Ovejas was estimated at 10,621 cases per 100,000 inhabitants, while in Corozal the attack rate was 1640 cases per 100,000 inhabitants (Table 1). Monthly incidence rates of Chikungunya (Table 1, Fig. 2) also showed a higher frequency of cases in Ovejas, which was noticeable during the months of September, October and November. In the months of December and January the incidences in the two towns were similar and descending, showing the end of the epidemic period.

**Table 1:** Monthly and cumulative incidences rates of Chikungunya in towns of Ovejas and Corozal

	Ovejas				Corozal			
	Cases		Population	Rate/100000 inhabitans	Cases		Population	Rate/100000 inhabitans
Months	#	%			#	%		
September	203	16.0	11947	1699.2	29	3.5	51157	56.7
October	530	41.8	11744	4512.9	82	9.8	51128	160.4
November	390	30.7	11214	3477.8	197	23.5	51046	385.9
December	137	10.8	10824	1265.7	461	54.9	50849	906.6
January	9	0.7	10687	84.2	70	8.3	50388	138.9
Total	1269	100	11947	10621.9	839	100	51157	1640.0

**Fig. 3:**
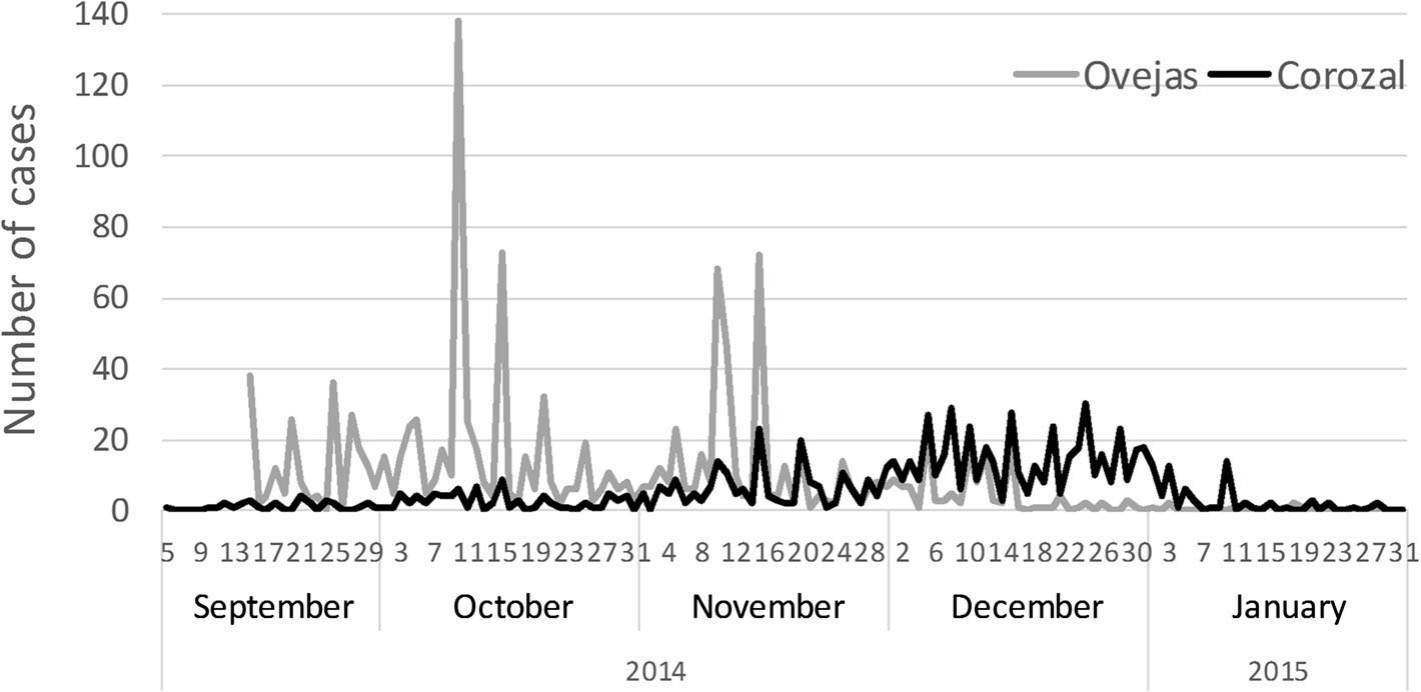
Epidemic curves of chikungunya in the towns of Corozal and Ovejas

The epidemic curves of Chikungunya in Corozal and Ovejas are shown in Fig. 3. A high rate of positioning and spread with high variation was observed in Ovejas, and a slow rate with greater stability in Corozal; the decline of the outbreak in the two towns was evidenced by the month of January. The duration of the epidemic in the two towns did not exceed 5 months.

### Survival to Chikungunya infection

**Fig. 4:**
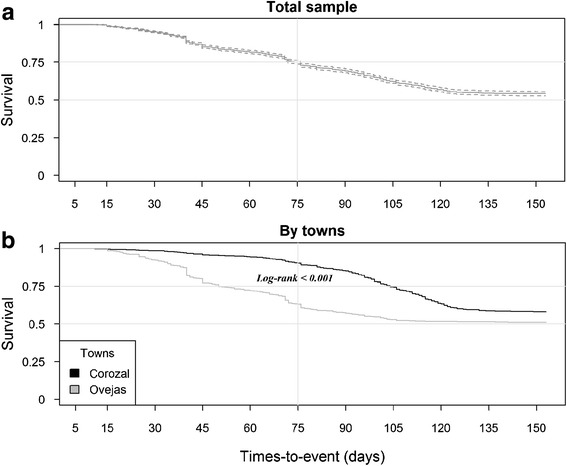
Survival curves for residents affected for chikungunya in Corozal and Ovejas. **a** Analysis of the total sample. **b** Comparative analysis between the towns

The survival curves generated by the Kaplan-Meier estimator for residents within affected households in the two towns are shown in Fig. 4. The survival curve with the total number of data sampled in two populations (Fig. 4a) showed by the middle of the study period (75 days), a survival probability of 0.75 (CI = 0.738–0.763) and at the end of period (150 days), the proportion fell to 0.54 (CI = 0.528–0.556). After 120 days, a stable horizontal slope was observed, indicating the end of the epidemic period.

When comparing the data from the two populations (Fig. 4b), survival curves showed different features. At 75 days, a marked reduction in the survival in Ovejas was observed (0.632, CI = 0.614–0.651); in contrast with the trend observed in Corozal (0.904, CI = 0.891–0.917). However, at the end of the epidemic period the two curves stabilized their slopes, continuing horizontal, parallel and close to a probability of 0.54.

The log-rank test showed high level of statistical significance (*X* ^2^ = 94.6, 1fd, *p*-value = 0.000) between the two curves, evidencing a different survival evolution to Chikungunya among the two populations.

### Socioeconomic factors and preventive measures

The results of the survey, which was applied to 516 households affected by Chikungunya in the town of Ovejas and 458 households affected in the town of Corozal are shown in Table 2. The table describes the number and the percentage of households with confirmation of the factors related to the disease, the application of preventive measures and the predisposing factors for proliferation and attack from vectors; Additionally, we present the measures of effect (risk ratio) of these variables for the two towns. High family income (> $USD 269, January/2015), mosquito fogging, mosquito repellent, garbage collection service, fibber cement roof and cement wall are all factors that demonstrated protection in dwellings studied of Corozal. In contrast, health expenditures, medical attention and stored water were variables that indicated risk in dwellings studied of Ovejas. For its part, the variables hospitalization, wire gauze windows, bed net and aqueduct supply were not determined to have significant differences between the two areas.

**Table 2:** Households in the towns of Ovejas and Corozal and its association with the epidemic of Chikungunya

Variables	COROZAL		OVEJAS		Risk ratio	95% CI		*p*-value
	*n*	%	*n*	%		Lower	Upper	
High family income	165	34.2	105	20.3	0.713	0.628	0.808	0.000
Health expenditures	351	72.8	504	97.7	2.232	2.031	2.452	0.000
Medical attention	291	60.4	363	70.4	1.248	1.098	1.418	0.001
Hospitalization	60	12.5	52	10.1	0.889	0.738	1.071	0.236
Mosquito fogging	221	45.9	0	0.0	0.336	0.304	0.371	0.000
Mosquito repellent	113	23.4	87	16.9	0.818	0.710	0.944	0.009
Mosquito net	10	2.1	19	3.7	1.413	0.852	2.343	0.131
Wire gauze	6	1.2	7	1.4	1.047	0.580	1.890	0.876
Aqueduct network	482	100.0	516	100.0	- - -	- - -	- - -	- - -
Stored water	255	52.9	512	99.2	2.956	2.670	3.272	0.000
Garbage Service	482	100.0	283	54.8	0.000	Undefined		0.000
Fiber cement roof	401	83.2	232	45.0	0.350	0.287	0.428	0.000
Cement wall	450	93.4	423	82.0	0.497	0.366	0.674	0.000

## Discussion

This is the first socioeconomic and demographic study that compared two populations in the Colombian Caribbean that have been severely affected by the chikungunya virus. In a previous study in 2014 that included both rural and urban areas, the department of Sucre was reported to have the second highest incidence of CHIKV in Colombia, with 14,741 cases and an attack rate of 1748 cases/100,000 inhabitants. Within Sucre, Ovejas had ranked second with a cumulative incidence of 4659 cases/100,000 inhabitants and Corozal had the sixth position with a cumulative rate of 2179 cases/100,000 inhabitants [22].

While the present study was exclusively focused on the urban area of the town of Ovejas and Corozal, the presentation of the disease followed the expected trend reported by the previous study [22]. Still, the attack rate by CHIKV in Ovejas (10,621/100,000 inhabitants) was 10 times higher than that observed in Corozal (1640/100,000 inhabitants). This could be evidence of serious problems in populated areas, including the urbanization of *Aedes aegypti* [23] and poor public health control measures. The attack rate by CHIKV in both cities was high, despite the fact that the outbreak took place in a dry season influenced by the el Niño phenomenon.

A higher frequency of occurrence, higher variability and higher speed of accommodation and spread of the virus was observed in Ovejas when comparing the two epidemic curves. Therefore, it can be asserted that the evolution of the epidemic process was different in the two urban areas. Additionally, the significance obtained from the log-rank test showed a better evolution in survival to CHIKV in Corozal; at 75 days the survival rate was 27.2% higher than that observed in Ovejas. Furthermore, the relative risk represented a protection factor for the inhabitants of Corozal, exhibiting 1.16 times less likely of contracting Chikungunya in comparison with the inhabitants of Ovejas.

Despite the differences in variability, incidence and survival analysis in the two towns, the decrease of the outbreak of Chikungunya took place approximately at 120 days, as it was observed both in the joint estimation of survival and in the separate estimate in the two towns. Previous studies conducted by the National Health Institute of Colombia showed a similar presentation [24, 25].

Socioeconomic factors and preventive measures are determinant in the rate of installation and spread of infectious diseases [26]. When the causal agent, vector and climatic conditions are similar between neighbouring communities, the differences in the evolution of the epidemic process can only be explained by their different socioeconomic conditions and their preventive measures.

Because the epidemiological questionnaire assessed the socioeconomic aspects and prevention against Chikungunya in the surveyed households and it specifies the time until the presentation of the event, no additional information is available in the residents to be associated through the Cox proportional hazards model that are normally applied in conjunction with the survival analysis [27]. However, public health aspects studied in households were able to identify either protective factors or risk factors for Chikungunya in the two towns that could be related with the best or worst epidemiological presentation of Chikungunya in the studied populations.

The two towns had water supply service; however, all the studied houses in Ovejas and the half houses in Corozal, they often stored water in tanks, a consequence of the intermittence or irregular water supply by aqueduct service. From a preventive point of view, the higher rate of storage water in tanks, the higher accumulation of wastes, the low frequency of applied insecticide spray and the low use of repellents by people in the urban area of Ovejas, produced the favourable conditions for the proliferation of mosquitoes and for the transmission of Chikungunya. Additionally, the deficient structural housing conditions allows for easier entry of mosquitoes. Finally, the high number of families with low income in Ovejas is a fundamental factor associated with a high health vulnerability.

Overall, better sanitary conditions were observed in Corozal than in Ovejas. These results agree with the official socioeconomic characterization defined by the National Administrative Statistics State of Colombia [19] through which the urban and rural area of Corozal shows better socioeconomic conditions than the municipality of Ovejas.

## Conclusions

The dynamics of the temporal distribution of CHIKV is influenced by socioeconomic and preventives factors. Precarious socioeconomic conditions, such as inefficient water supply and waste collection services, may be determining factors in the proliferation of CHIKV. The survival analysis proved to be an appropriate method to analyze the temporal presentation of CHIKV and can be applied to other infectious diseases transmitted by vectors with a similar performance, as is the case of the Dengue and ZIKA arboviruses. The timely intervention of outbreaks with an analysis such as the one presented in this study, may be a useful tool for an efficient response towards the control of the epidemic outbreaks that may occur in the future.
